# Vertical transmission in field-caught mosquitoes identifies a mechanism for the establishment of Usutu virus in a temperate country

**DOI:** 10.1038/s41598-025-09335-x

**Published:** 2025-07-12

**Authors:** Mirjam Schilling, Becki Lawson, Simon Spiro, Madhujot Jagdev, Alexander G. C. Vaux, Robert C. Bruce, Colin J. Johnston, Anthony J. Abbott, Ethan Wrigglesworth, Paul Pearce-Kelly, Andrew A. Cunningham, Jolyon M. Medlock, Nicholas Johnson, Arran J. Folly

**Affiliations:** 1 Virology Department, Animal and Plant Health Agency, Woodham Lane, Addlestone, KT15 3NB Surrey UK; 2 Institute of Zoology, Zoological Society of London, Regent’s Park, London, NW1 4RY UK; 3 Wildlife Health Services, Zoological Society of London, Regent’s Park, London, NW1 4RY UK; 4 Medical Entomology and Zoonoses Ecology Group, UK Health Security Agency, Salisbury, SP4 0JG UK; 5 Zoological Society of London, London, NW1 4RY UK

**Keywords:** Flavivirus, *Culex pipiens*, Vertical transmission, Emerging infectious disease, Mosquito-borne disease, Overwintering, Diapause, Viral transmission, Viral vectors

## Abstract

Usutu virus (USUV) is an emerging zoonotic flavivirus in Europe and the first zoonotic mosquito-borne virus to be confirmed in animal hosts in the United Kingdom (UK). Repeated detection of USUV in the years following its initial detection in 2020 indicated that the virus is overwintering in the UK and should now be considered endemic within southeast England. Surveillance of avian hosts and mosquito vectors has been insufficient to elucidate the mechanism by which USUV has persisted through temperate winters. Through enhanced targeted vector surveillance at the index site between 2021 and 2024 inclusive, we detected USUV RNA in host-seeking adult female *Culex pipiens* s.l. as well as in adults reared from field-caught larvae. This is, to our knowledge, the first description of vertical transmission of USUV in an arthropod vector that should consequently be considered a viable mechanism for the persistence of USUV in temperate areas.

**Supplementary Information:**

The online version contains supplementary material available at 10.1038/s41598-025-09335-x.

## Introduction

Global changes in climate are increasing the likelihood of vector-borne disease outbreaks^1,2^. This is especially true for temperate regions that were once considered safe from the establishment of diseases from the tropics or sub-tropics^3^. Usutu virus (USUV, family: *Flaviviridae*), first detected in South Africa in 1959^4^, has spread across mainland Europe in recent decades^5^ and was first confirmed in the United Kingdom in 2020^6^. The virus was repeatedly confirmed in Greater London in the 3 years following the initial outbreak, primarily in blackbirds (*Turdus merula*) that were found dead, or euthanised, due to morbidity for welfare reasons and submitted for post-mortem examination. Molecular clock analysis of USUV sequences obtained from infected birds in England across multiple years revealed that they shared a most recent common ancestor, indicating that the virus persists by overwintering in the UK^7^. In 2023, USUV was detected in southeast England outside Greater London for the first time in an infected juvenile blackbird^8^. The recovered genomic sequence was closely related to the Greater London USUV detections, demonstrating previously unrecorded geographic expansion since the 2020 USUV outbreak. Therefore, USUV may now be considered endemic in southeast England.

Typically, USUV infections affect passerines (especially blackbirds) and Strigiformes^8,9^. Indeed, blackbird populations in Greater London appear to have declined by up to 39% since the emergence of USUV in 2020^9,10^, indicating that the virus may have had a rapid, negative impact on susceptible hosts. Although human infection is most commonly asymptomatic, multiple cases of short-term neurological disease have been reported^11^. Data from Italy reveal that seropositivity in asymptomatic humans can be as high as 7% (cerebrospinal fluid and serum samples collected between 2008 and 2011 in Modena)^12^ or 46% (blood donors in the Lombardy region)^13^ in certain areas, compared to 1% in blood donors in the Po River Valley in northern Italy reported between 2014 and 2015^14^. These data show that USUV can infect healthy people, with those in areas of higher viral prevalence where bridge vectors are present, such as forestry workers (18% seroprevalence in northern Italy in 2014–2015)^14^ or bird ringers (23.6% seroprevalence in the Netherlands 2021), with increased risk of exposure^15^. While there have been no confirmed cases of USUV infection in people in the UK to date, understanding how USUV persists in temperate areas is a key step to inform our understanding of the risk this virus may pose to animal and public health.

As flaviviruses exist in an enzootic cycle, both vector and host likely contribute to the establishment of endemicity in any given area. In order to persist in temperate areas, mosquito-borne viruses must overwinter in either hosts (with the maintenance or recrudescence of virus infection), or vectors to facilitate autochthonous transmission when conditions become permissive. Given that infected birds can rapidly clear USUV infection through mounting a sufficient antibody response^16,17^, or succumb to the infection^8^, it is unlikely that USUV would persist in avian hosts throughout temperate winters. Therefore, it is likely that mosquitoes provide a mechanism for the virus to persist. Either maintenance of infection in diapausing adult mosquitoes or vertical transmission to larvae could be required for overwintering viral persistence in temperate areas. However, it has proven challenging to detect USUV in wild diapausing mosquitoes or in developing progeny^18^. During the course of our study, virus detection in overwintering female *Culex torrentium* in Poland^19^, and in *Culex pipiens/torrentium* collected in the Netherlands have been published^20^.

To improve our understanding of the drivers of USUV establishment, alongside passive surveillance in wild birds^8^, we conducted targeted vector surveillance at the site of the initial USUV outbreak in 2020 and where USUV had been detected annually, from 2021 to 2024 inclusive. This comprised (1) Adult mosquito surveillance to improve understanding of the virus circulation and bloodmeal analysis to study transmission networks. (2) Collecting overwintering mosquitoes to elucidate mechanisms for virus persistence and (3) Sampling mosquito larvae to investigate potential for vertical transmission. This approach enabled an increase in viral RNA detection across mosquito life stages, including evidence for vertical transmission, and indicates that *Culex pipiens* s.l. mosquitoes are involved in the persistence of USUV in temperate areas.

## Materials and methods

### Enhanced vector surveillance

To improve our understanding of the contribution of the vector population to the persistence of USUV in the UK, enhanced surveillance was conducted. Samples comprised of (1) adult mosquitoes, (2) overwintering mosquitoes, and (3) mosquito larvae.

1. Adult female mosquitoes were collected in 2022 and 2023 from ZSL London Zoo, the 2020 index site, using BG-Sentinel 2^®^ traps (Biogents AG, Regensburg, Germany) baited with BG-Lure (Biogents AG) (see Supplementary Tables 1 and 3). Where possible, captured mosquitoes were collected weekly (2022: Sept; 2023: Jul – Sept) and kept in a -20 °C freezer prior to the identification prior to further analysis.
2. Overwintering mosquitoes were collected from November to February at ZSL London Zoo each year, 2021 to 2024 inclusive, in an attempt to elucidate the mechanism of USUV persistence where temperate winters preclude year-round virus transmission (see Supplementary Tables 1 and 3). Sites for collection had been identified by members of staff at the zoo and included a concrete pump room (location identified using what3words: rescue.career.tubes) and a concrete storage room (location identified using what3words: popped.usual.spot). A mechanical aspirator was used to collect resting adults. For the last collection in winter 2022/23, and all collections in winter 2023/24, mosquitoes were taken to an insectary at APHA Weybridge and held for 3–4 weeks at 25 °C and 50% humidity (12:12 light cycle), and emerged adults were kept under the same conditions (see paragraph below). This was done to end dormancy and increase metabolic activity, which might reactivate viral replication, facilitating detection using RT-qPCR. A similar approach has been successful for West Nile virus (WNV)^21^ and St. Louis encephalitis virus (SLEV)^22^ detection from overwintering *Culex pipiens.* Captive mosquitoes were checked daily. Any found dead were recorded and stored at − 80 °C for later molecular analysis. The first winter collections in 2022 and 2023 did not yield any USUV-positive mosquito pools when the mosquitoes were kept in the laboratory for 4 weeks. Due to this timeframe resulting in no USUV RNA recorded from mosquitoes in a location shown to have USUV circulation, we hypothesised that mosquitoes might naturally clear the infection over time after diapause. We therefore further adapted our protocol and sampled mosquitoes at 10 days and 3 weeks post-collection for the mosquitoes captured in December 2023 and January 2024.
3. To identify if USUV vertical transmission occurs, in 2023 wild mosquito larvae (all first (L1) and second stage (L2) larvae) were collected from June to August 2023 inclusive, at ZSL London Zoo (see Supplementary Tables 2 and 3). We targeted standing water pools and additionally established oviposition traps (darkened plastic containers filled two-thirds with water) in areas where birds in the zoological collection had seroconverted following autochthonous USUV transmission^16,17^. Open traps were set up using fresh tap water mixed with a small proportion of standing water found on site to mimic more realistic habitat conditions. Larvae were collected from the water pools and traps with Pasteur pipettes and aquatic nets and transported to the laboratory in Falcon tubes containing a small amount of the larval habitat water. In the laboratory, the larvae (together with the remaining larval habitat water in the Falcon tube) were transferred to a new larval tray containing fresh water and reared to adulthood in an insectary (environmental conditions as above). Adult mosquitoes were checked daily and kept alive for up to 4 weeks before being euthanised (by freezing them at -80 °C) for molecular analysis. During rearing, any adult mosquitoes found dead were recorded and stored at -80 °C prior to later molecular analysis.

A summary of all trapping locations and types of traps can be found in Supplementary Tables 3 and Supplementary Fig. 1. Trap locations were documented using what3words (https://what3words.com). The map was generated using QGIS Maidenhead v3.36.1 (https://qgis.org/).

### Mosquito identification by morphology

All mosquitoes collected were identified morphologically to the lowest taxonomic certainty following the taxonomic keys delineated by Snow^23^. Since the females of *Cx. pipiens* (biotypes *pipiens pipiens* and *pipiens molestus*) and *Cx. torrentium* cannot be differentiated with morphological characteristics^24^, our collection may likely have included *Cx. pipiens* and *Cx. torrentium* (for readability hereafter referred to as *Cx pipiens* s.l.). Studies show that all three taxa are present in Greater London, with the vast majority belonging to *Culex pipiens* s.s^25^.

### RNA extraction and Usutu virus screening

Mosquitoes were homogenised in pools of ≤ 10 in 300 µl tissue culture medium using the Qiagen TissueLyser II with 5 mm stainless steel beads (both Qiagen, Manchester, UK) and centrifuged (10,000 rpm/10 min). Total RNA was extracted from 250 µl of the supernatant using TRIzol^®^ (Invitrogen, Life Technologies Limited, Paisley, UK). The precipitated RNA was resuspended in 20 µl nuclease free water and subjected to an USUV-specific RT-qPCR^26^. Virus isolation was attempted from homogenate (stored at -80 °C) of PCR-positive mosquito pools in Vero cells in a BSL3 laboratory at the APHA.

### DNA barcoding

In order to understand potential transmission networks, we attempted to retrieve cytochrome c oxidase I (COX1) sequences of vertebrate hosts from mosquito bloodmeals. Sixteen blood-fed wild mosquitoes caught in September 2022 were processed individually, and DNA was extracted from the blood meal (removed from mosquito using forceps to apply pressure to the abdomen) using the DNeasy Blood & Tissue Kit (Qiagen) according to the manufacturer’s instructions. A 658 bp region located at the 5’ end of the COX1 gene was amplified by PCR as previously established^9,27^. PCR products were visualised on a 1.5% agarose gel, and samples of the correct band size were submitted for Sanger sequencing.

### Next generation sequencing and phylogenetic analysis

Mosquito pools that were positive in the USUV-specific RT-qPCR were subjected to next generation sequencing in an attempt to recover a complete virus genome. Sequencing libraries were prepared using the Nextera XT kit (Illumina, Cambridge, UK) and analysed on a NextSeq sequencer (Illumina, Cambridge, UK) with 2 × 150 base paired-end reads. Consensus sequences were generated by using a combination of the Burrows–Wheeler Aligner v0.7.13 and SAMtools v1.9 with a representative USUV genome (MW001216) as a scaffold. Two samples, an adult mosquito pool and an adult reared from a larva, both collected in 2023, produced approximately 5 kb of sequence data that aligned to a USUV reference genome (11 kb - GenBank accession number: MW001216). These sequences clustered with 15 confirmed USUV Africa lineage 3.2 isolates from across mainland Europe using MAFFT v7.471. The resulting alignment was imported into BEAST (v1.10.4) and a Bayesian phylogenetic tree was produced using the GTR + I nucleotide substitution model and 10,000,000 Markov chain Monte Carlo generations. Log files were analysed in Tracer v1.7.1 to check the effective sample size, and a 10% burn-in was included (TreeAnnotator v.1.10.4) before being visualised and annotated in FigTree v1.4.4.

## Results

### Enhanced vector surveillance increases USUV detection in mosquitoes

**Fig. 1:**
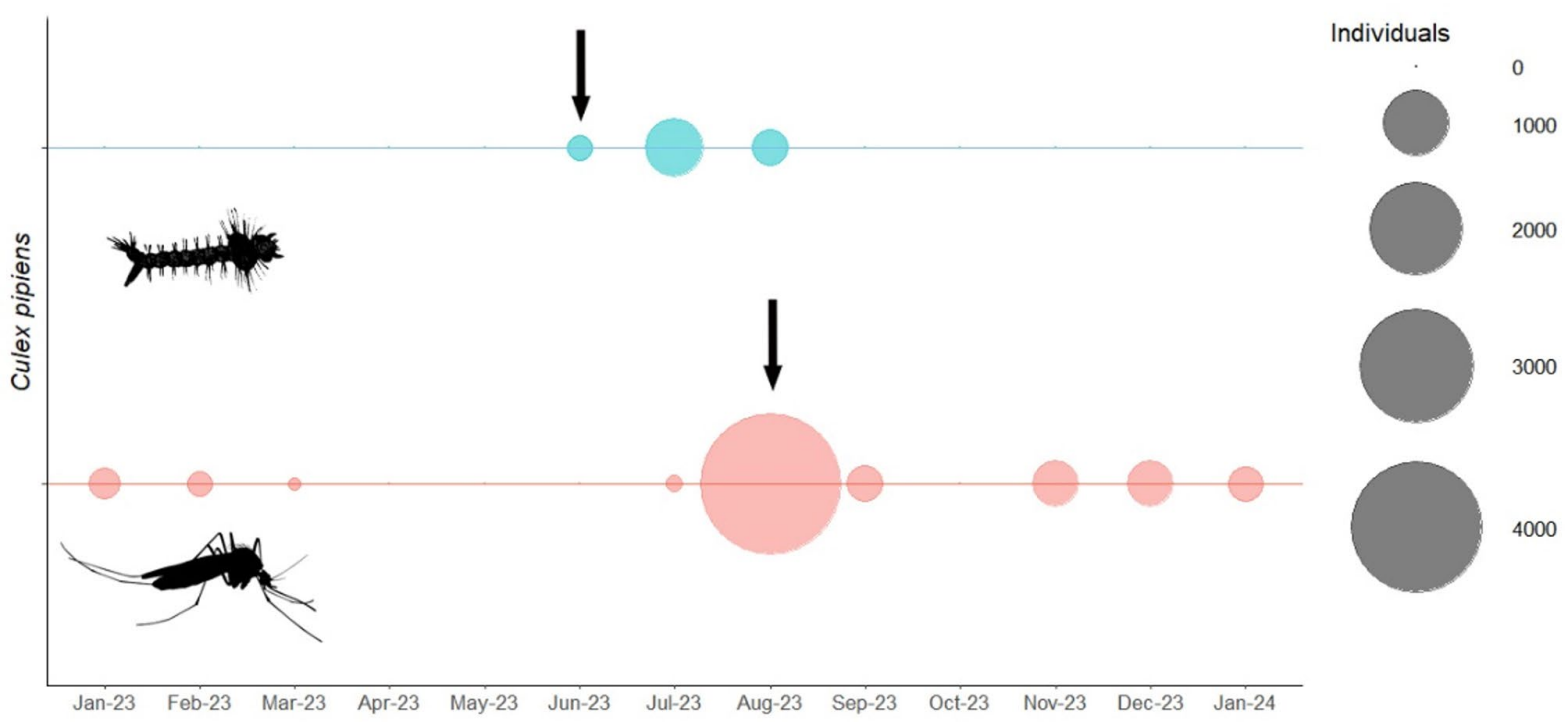
Usutu virus spreads within the *Culex pipiens* s.l. population through vertical transmission. Bubble plot representing the number of adult *Cx. pipiens* s.l. (red) and the number of adult *Cx. pipiens* s.l. reared from larvae collected in the field (blue) between January 2023 and January 2024 inclusive. Arrows mark detections of USUV RNA by RT-qPCR (*n* = 6 adults, *n* = 1 reared from larvae).

During the mosquito active season in 2022 and 2023, we trapped 156 and 5433 adult mosquitoes, respectively, the majority (92.0%) of which were *Cx. pipiens* s.l. (Fig. 1, Supplementary Table 1). Of 55 pools tested from 2022, two pools (3.6%), collected in September, were positive for USUV RNA by RT-qPCR. In 2023, six of 540 pools (1%) tested positive for USUV RNA by RT-qPCR. All six positive pools derived from mosquitoes collected in August. Based on our pool size of 10 these represent minimum infection rates of 0.36% and 0.1%. From one of the 2023 pools we were able to recover a 5088 bp section of the USUV genome for phylogenetic analysis (see below). All pools that tested positive for USUV RNA were *Cx. pipiens* s.l. (Supplementary Table 1). However, we were not able to isolate the virus from the USUV positive samples, which had been stored for extended duration at − 20 °C. Additionally, we investigated host-feeding preferences of the mosquitoes present at the zoo, as this is an important aspect of the role of *Culex* communities in the local USUV persistence. It highlights potential transmission cycles in zoological collections and which animals may be at risk of USUV transmission. We identified a total of 16 blood-fed female mosquitoes trapped during September 2022. Blood meal samples from *Cx. pipiens* amplified avian COXI sequences for four of the 16 blood meal samples, likely limited by poor sample quality as mosquitoes were not kept in a cold chain: those identified were from a southern caracara (*Caracara plancus*) (*n* = 1), grey-capped emerald dove (*Chalcophas indica*) (*n* = 1), and scarlet ibis (*Eudocimus ruber*) (*n* = 2). These are zoological collection birds housed in outdoor enclosures at ZSL London Zoo and are therefore likely to be accessible to host-seeking, primarily ornithophagic mosquitoes. No USUV-associated disease has been observed in these collection birds.

### Trialling an adapted protocol to detect USUV in diapausing females

A total of 1950 diapausing females, the majority *Cx. pipiens* s.l. (99.5%), were collected during the winters 2021/22, 2022/23, and 2023/24 (Supplementary Table 1). All of the 40 mosquito pools collected in winter 2021/22, 63 pools collected in winter 2022/23, and 139 collected in winter 2023/24 (total pools *n* = 242 pools) were negative for USUV RNA by RT-qPCR (Supplementary Table 1).

#### Vertical transmission contributes to USUV persistence in *Cx. pipiens* s.l.

**Fig. 2:**
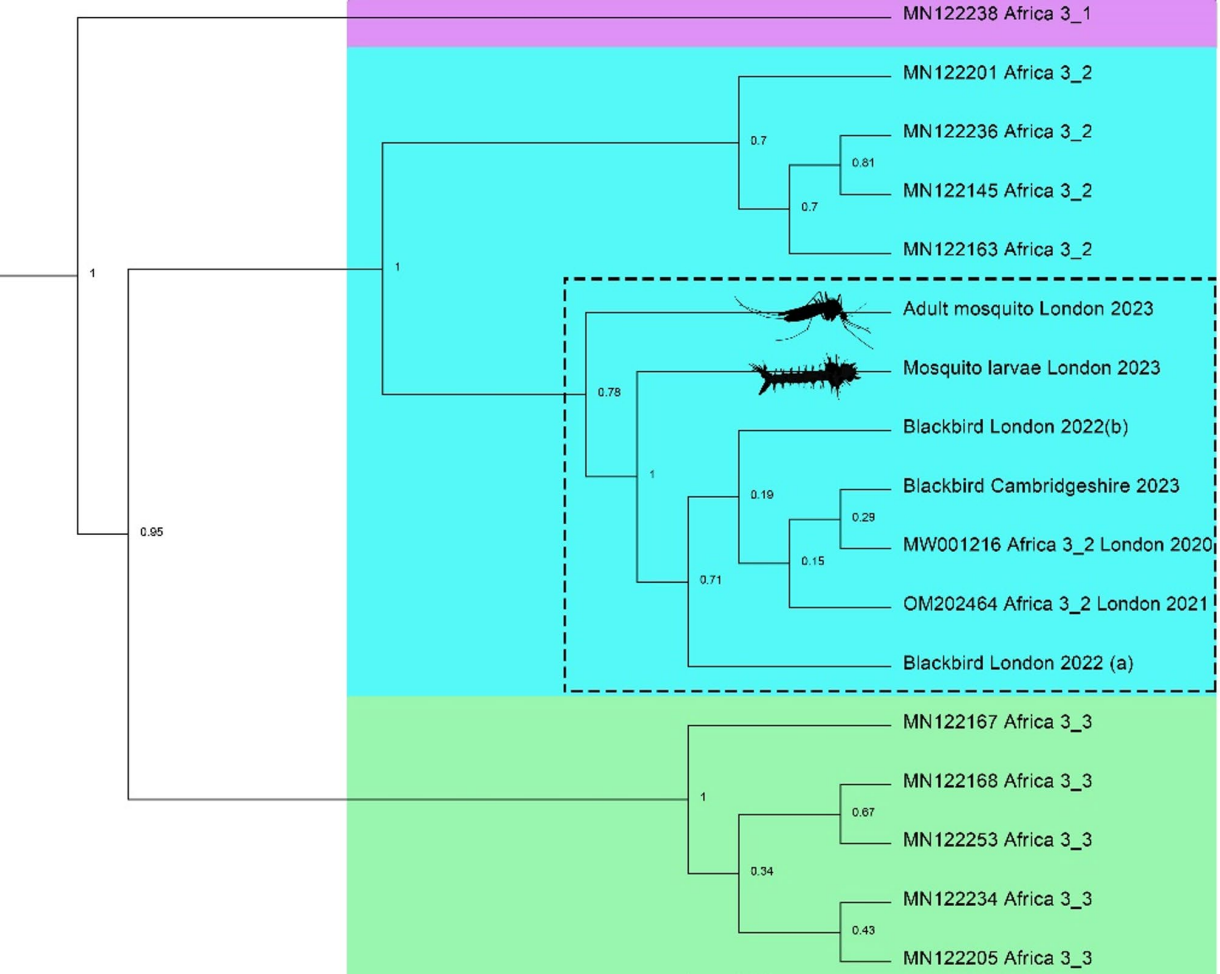
Usutu virus (USUV) sequences from mosquitoes (*Culex pipiens* s.l.) cluster with sequences of isolates from wild birds in England. Bayesian phylogenetic analysis of USUV sequence data (approximately 5 kb) retrieved from an adult mosquito pool as well as two adults reared from larva in 2023. The sequences were aligned with 15 USUV Africa lineage isolates from across mainland Europe. Node labels represent posterior probabilities and accession numbers are shown.

We reared 1297 adults from wild larvae (L1 and L2) collected in June 2023 (Fig. 1, Supplementary Table 2). One pool (*n* = 2 adults) tested positive for USUV RNA (Fig. 2), indicating vertical transmission of the virus. The positive pool contained two male *Cx. pipiens* s.l. that had died within 7 days of emergence and whose larvae were originally collected from a central location at ZSL London Zoo (what3words location: Gravy.Riots.Bugs). We were unable to isolate virus from homogenate in Vero cells, but next generation sequencing of the positive pool recovered a 5210 bp sequence which mapped to USUV lineage Africa 3.2 (GenBank accession number: MW001216, average read depth = 781). Phylogenetic analysis of a 5088 bp region shows that both the sequence obtained from vertical transmission and the adult mosquito sequence (described above) cluster within the USUV lineage Africa 3.2 UK clade (Fig. 2).

## Discussion

In addition to facilitating the detection of USUV RNA in wild-caught adults, our enhanced mosquito surveillance detected USUV RNA in adults which had emerged from wild-caught larvae. This indicates that USUV was likely transmitted vertically from an infected, gravid, female. To our knowledge, this is the first time vertical transmission has been identified for USUV in an arthropod vector. Vertical transmission of two closely related members (WNV and SLEV) of the Japanese encephalitis virus serogroup has been reported previously, supporting the likelihood of our findings^28,29^. However, we cannot fully exclude that the larvae were infected horizontally through the water at the habitat site. For WNV, the possibility of infection of mosquito larvae through WNV-positive excreta has been experimentally demonstrated^30^. However, the efficiency of this process was limited by the low stability of viral particles in water. Additionally, larvae were less susceptible than pupae. Similarly, transovum transmission has been shown to be a viable, if highly inefficient, route of vertical transmission of viruses in mosquitoes, even in laboratory-based experiments, with controlled, high titre inoculations, and high confirmed viral infection prevalence in female mosquitoes^31^. No studies on transovum transmission have been performed on USUV-infected *Cx. pipiens*. In contrast, in field data with very low prevalence of infection in adult mosquitoes, transovarial transmission is considerably more likely (and is documented as having a much higher efficiency^32^). In line with previous studies investigating vertical transmission of other flaviviruses from field samples with low prevalence, we believe transovarial transmission is the most likely mechanism for our results^33,34^.

Even under apparent high virus occurrence at the index site, indicated by the recovery of dead wild passerines infected with USUV and seroconversion of multiple birds in the zoological collection^7^, our study found the prevalence of USUV RNA detectable by RT-qPCR was low in the mosquito community. This is in line with previous results from earlier mosquito surveillance at the index site in 2021^7^ and similar studies performed in mainland Europe where USUV is circulating^35^. It is unclear if these results are caused by a genuine low prevalence of USUV in the vector population or if virus is maintained at titres within the insect host below the level of detection of our RT-qPCR assay. However, our detected prevalence of 0.1% (2023) and 0.36% (2022) in mosquito pools (comparable to the minimum infection rates of 0.2%, as reported by^35^) demonstrates the strength of our targeted surveillance efforts and provides a robust data set to investigate virus dynamics within the local mosquito population. Studies on related flaviviruses using next generation sequencing technologies report difficulty in detecting viral RNA without targeted amplification of flavivirus RNA prior to sequencing, even in areas with known high virus prevalence, suggesting that virus titres in vectors are naturally low^36^. Our investigations of USUV, which targeted the different stages (collected as larvae and adults, and tested as adults) of the mosquito life cycle, help to elucidate the contributions of arthropod vectors to the persistence and endemicity of USUV in temperate areas.

We detected USUV RNA in two pools of adult mosquitoes collected in September 2022 and six pools in August 2023, the period of peak abundance of adult mosquitoes in Greater London^7,37^. Combining vector abundance data with USUV prevalence will help to identify areas and times of the year when there is an increased risk of virus exposure, to raise awareness amongst medical and veterinary communities, and target mitigation measures to safeguard health, such as deploying targeted mosquito control. Additionally, our blood meal analyses show that *Cx. pipiens* is an opportunistic, primarily ornithophagic mosquito, meaning that a diverse range of birds may inadvertently be exposed to virus. This is in line with confirmed USUV detections in the UK and across mainland Europe as well as seroconversion following autochthonous USUV transmission, identified in a range of birds held in the zoological collection at ZSL London Zoo^7,9^. Consequently, it is likely that species of birds in addition to wild passerines may be contributing to the enzootic cycle in a given area.

While USUV is emerging across Europe and is considered endemic in many countries, detection of overwintering flaviviruses in diapausing mosquitoes is rarely recorded^18,38^. Since mRNA expression during diapause is reduced and a transcriptional program leads to metabolic repression^39,40^, it is likely that viral RNA genome is maintained at low titres in mosquito cells until conditions for replication become favourable. Therefore, our methodology opted to rear overwintering females in the laboratory under optimum summer conditions for up to four weeks after field sampling. However, these conditions did not result in detection of USUV RNA, even after readjusting the protocol with a group of females being tested as early as 10 days after collection. Therefore, it is likely that either there was no USUV RNA or no active infection in the mosquito community we sampled, or that effective USUV replication would have required longer than our protocol allowed^41,42^. Future studies might also attempt to bloodfeed mosquitoes after diapause to investigate whether this initiates virus replication. If successful, this might imply that in the field, viruses emerge in the first generation of larvae rather than in the overwintering mosquito. Infection prevalence for the overwintering of a related flavivirus, WNV, has been published to be as low as 0.016% and might be similar for USUV^43^. Our recommendation is that laboratory-based experiments investigating the ability of arboviruses to persist through, and replicate after, diapause would provide a deeper understanding of USUV vector infection dynamics. However, we note that our overwintering survey was conducted in a restricted geographic area in central London, and more mosquito hibernacula in a wider area should be investigated.

There are likely multiple drivers for the establishment of mosquito-borne pathogens in new areas. Vertical transmission in arthropod vectors, generally observed at relatively low levels in field samples, has been implicated as a potential persistence mechanism for flaviviruses during periods of unsuitable climatic conditions or in the absence of vertebrate hosts^44,45^. Through our enhanced surveillance, we detected USUV RNA in adult mosquitoes that had emerged from field-caught larvae. To our knowledge, this is the first time data supporting vertical transmission has been reported for USUV in arthropod vectors. As vertical transmission allows virus spread within the mosquito population independent of any host activity, this may be a key mechanism for the establishment of this virus in temperate areas. One could also speculate that our detection of vertical transmission of USUV in a larval pool in June is an indirect indication for overwintering, as these might have been the first larvae that developed from eggs laid after diapause^37^.

As some native UK mosquito species are competent vectors for USUV^46^, our results emphasise the need to better understand the enzootic cycle of USUV in the UK, with a particular focus on the involvement of mosquitoes in viral persistence. This is especially true if bridge vectors are involved in enzootic cycles, as this could have negative implications for public health. Our data implicate both adult mosquitoes and developing larvae in the establishment of endemicity in temperate areas. Since flaviviruses are set to continue to spread across Europe in the coming years^47^, our study highlights the value of enhancing vector surveillance to help inform disease ecology and epidemiology and to identify areas that warrant targeted mosquito control to minimise infection risk for humans and animals.

## Electronic supplementary material

Below is the link to the electronic supplementary material.

Supplementary Material 1

## Data Availability

The nucleotide sequences obtained in this study have been deposited in GenBank under accession numbers: PV625057 (larvae) and PV625058 (adult).
