# Supplementary material for: Vertical transmission in field-caught mosquitoes identifies a mechanism for the establishment of Usutu virus in a temperate country
Source: Sci Rep. 2025 Jul 12;15:25252. doi: 10.1038/s41598-025-09335-x (PMC12255745; doi:10.1038/s41598-025-09335-x)
Supplement: Supplementary file 1 — Supplementary Material 1 [file 41598_2025_9335_MOESM1_ESM.docx]

**Supplementary Tables**

Supplementary Table 1: Adult mosquitoes collected at the index site in Greater London, UK, October 2021 to January 2024 inclusive.

| **Year** | **Month** | **Culex pipiens s.s./Cx. torrentium trapped** | **Pools** | **USUV positive pools** | ***Culiseta annulata* trapped** | **Pools** | **USUV positive pools** |
| --- | --- | --- | --- | --- | --- | --- | --- |
| 2021 | October | 30 | 3 | 0 | 0 | 0 | 0 |
| 2021 | November | 40 | 4 | 0 | 0 | 0 | 0 |
| 2021 | December | 12 | 3 | 0 | 2 | 2 | 0 |
| 2022 | January | 171 | 26 | 0 | 2 | 2 | 0 |
| 2022 | September | 151 | 51 | 2 | 5 | 4 | 0 |
| 2022 | October | 2 | 2 | 0 | 1 | 1 | 0 |
| 2022 | November | 32 | 6 | 0 | 1 | 1 | 0 |
| 2022 | December | 43 | 7 | 0 | 0 | 0 | 0 |
| 2023 | January | 221 | 25 | 0 | 0 | 0 | 0 |
| 2023 | February | 143 | 21 | 0 | 0 | 0 | 0 |
| 2023 | March | 32 | 4 | 0 | 0 | 0 | 0 |
| 2023 | July | 61 | 11 | 0 | 0 | 0 | 0 |
| 2023 | August | 4600 | 460 | 6 | 390 | 39 | 0 |
| 2023 | September | 300 | 32 | 0 | 50 | 5 | 0 |
| 2024 | November | 482 | 54 | 0 | 3 | 3 | 0 |
| 2023 | December | 480 | 51 | 0 | 1 | 1 | 0 |
| 2024 | January | 278 | 29 | 0 | 1 | 1 | 0 |

Supplementary Table 2: Adult mosquitoes reared from larvae collected at the index site in Greater London, UK, in 2023.

| **Month** | **Adult *Culex pipiens* s.s.*/Cx. torrentium* emerged** | **Pools** | **USUV positive pools** | **Adult *Culiseta annulata* emerged** | **Pools** | **USUV positive pools** |
| --- | --- | --- | --- | --- | --- | --- |
| June | 137 | 53 | 1 | 2 | 1 | 0 |
| July | 789 | 83 | 0 | 29 | 13 | 0 |
| August | 304 | 66 | 0 | 36 | 14 | 0 |

Supplementary Table 3: Overview of traps and locations organised by collection type.

| **Location** | **No. of traps** | **Trap type** | **Type collected** | **What3Word** |
| --- | --- | --- | --- | --- |
| African (‘Safari’) Aviary (external) | 2 | Biogents trap | Host-seeking females | custom.eggs.trip, before.cope.cliff |
| African (‘Safari’) Aviary (external) |  | Resting collection | Host-seeking females | perky.heats.deeper |
| Mappins service area (internal) | 1 | Biogents mosquitaire trap | Host-seeking females | curiosity.lonely.tennis |
| Mappins service area (internal) | 1 | Biogents sentinel trap | Host-seeking females | scores.deeply.opera, |
| Mappins service area (internal) |  | Resting collection | Host-seeking females | agenda.score.drives |
| Penguins service area (external) | 1 | Biogents sentinel trap | Host-seeking females | crown.lend.gloves |
| Penguins service area (external) | 2 | Biogents mosquitaire trap | Host-seeking females | universally.easy.raves |
|  |  |  |  | jump.gates.ballots |
| Tiger Territory (external) |  | Resting collection | Host-seeking females | intend.plank.pocket |
| Vet Hospital service area (external) |  | Magnet trap | Host-seeking females | paper.glee.office |
| Colobus monkey pump room (internal) |  | Resting collection | Diapausing females | rescue.career.tubes |
| Mappins service area (internal) |  | Resting collection | Diapausing females | popped.usual.spot |
| African (‘Safari’) Aviary (external) | 3 | Open water pools | Larvae | flock.secret.files |
| Blackburn Building 1 (external) | 1 | Open water pools | Larvae | gent.pulled.sobs |
| Blackburn Building 2 (external) | 1 | Open water pools | Larvae | gravy.riots.bugs |
| Mappins service area (external) | 3 | Open water pools | Larvae | formal.blur.raves |
|  |  |  |  | marked.stroke.pound |
|  |  |  |  | about.filed.rivers |
| Northbank (external) | 2 | Open water pools | Larvae | garden.fairly.hero |
| Penguins service area (external) | 3 | Open water pools | Larvae | common.swear.items  total.repay.admiral  desks.darker. oval |
| Vet Hospital service area (external) | 2 | Open water pools | Larvae | birds.zoom.divide |
|  |  |  |  | issued.regime.cable |

**Supplementary Figures**


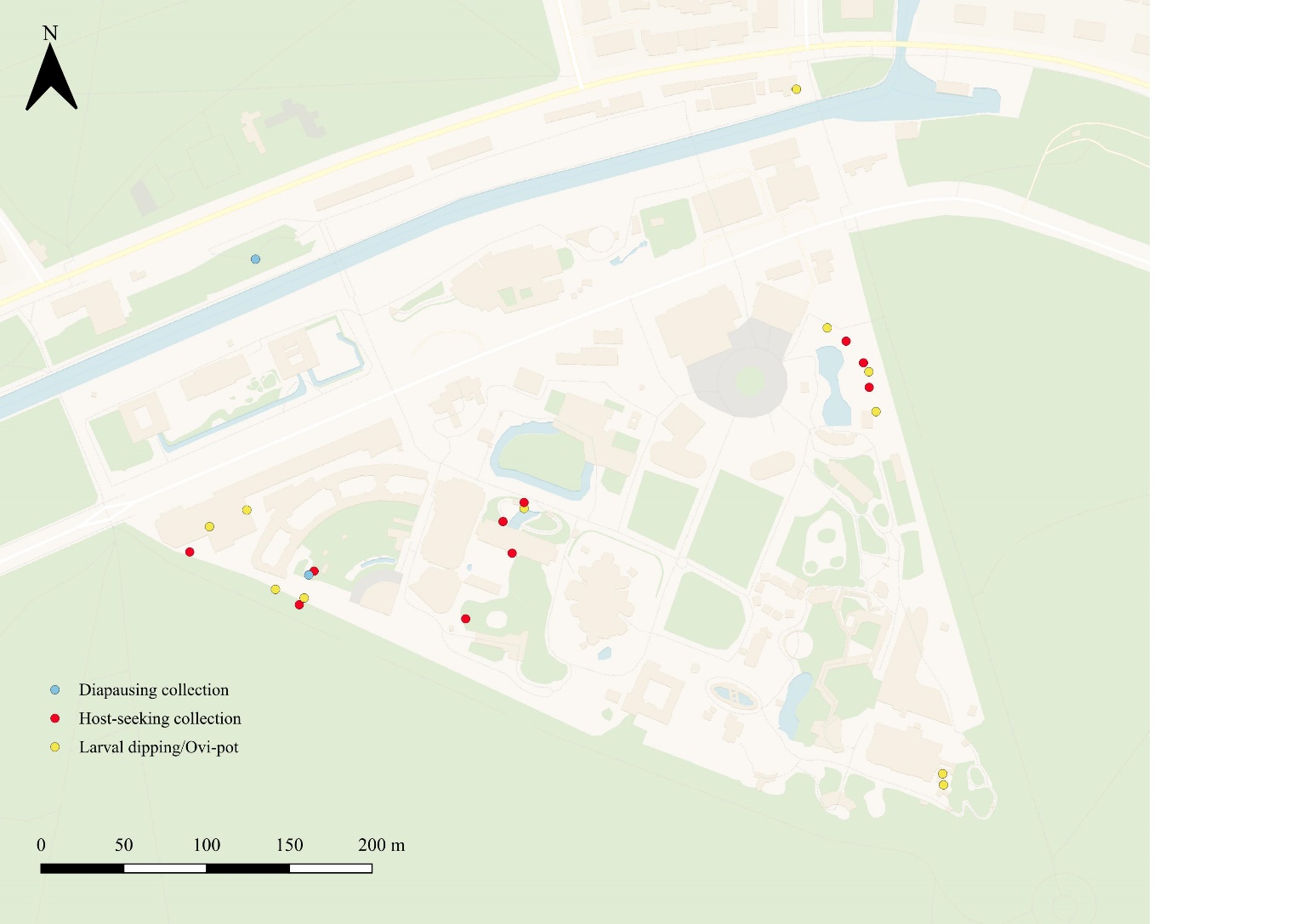


Supplementary Figure 1: Trapping locations at London Zoo. Trap locations are depicted according to the what3words coordinates in Suppl. Table 3 and colour coded depending on the type of collection. The map was created using QGIS (open access QIG software, v3.36.1 https://qgis.org/).
